# ﻿Female and male genital surface microstructures in shining leaf beetles (Coleoptera, Chrysomelidae, Criocerinae)

**DOI:** 10.3897/zookeys.1252.148379

**Published:** 2025-09-19

**Authors:** Yoko Matsumura

**Affiliations:** 1 Department of Systematic Entomology, Graduate School of Agriculture, Hokkaido University, 060-8589 Sapporo, Japan Hokkaido University Sapporo Japan

**Keywords:** Bursa copulatrix, criocerine beetles, endophallus, internal sac, scanning electron microscopy (SEM), sensilla, spermathecal duct

## Abstract

Female genital diversity and co-evolution of female and male genitalia have been increasingly unveiled, particularly through recent studies. These findings highlight the importance of genital coupling in genital studies. In this pilot study, I examined membranous elements of female and male genitalia that come into physical contact during copulation in five representative species of shining leaf beetles by employing stereomicroscopy and scanning electron microscopy. The female genital surfaces are largely smooth, but a thickened sensilla-bearing patch is present. In contrast, the male surfaces are at least partially covered with microprotrusions. I present distribution maps of these microstructures for all study species. By comparing the results with those of previous studies, I discuss their possible functions and future research directions in this field.

## ﻿Introduction

Female genital diversity remains less explored compared to male genitalia due to historically long-standing neglect of the female genital morphology (Ah-King et al. 2014; Orbach 2022). However, recent studies have unveiled a hidden diversity and co-diversification between the sexes (e.g., Ilango and Lane 2000; Rodriguez et al. 2004; Macagno et al. 2011; Yassin and Orgogozo 2013; Kotrba et al. 2014; Matsumura et al. 2014; Genevcius et al. 2017). These findings highlight the significance of understanding genital interactions.

Genital interactions have primarily been studied by employing instant fixation of pairs followed by dissection (e.g., Düngelhoef and Schmitt 2006; Flowers and Eberhard 2006; Matsumura and Akimoto 2009; Matsumura and Yoshizawa 2010), and recently micro-computed tomography (µCT) has often been employed as a non-invasive method to observe genital coupling (e.g., Schmitt and Uhl 2015; Matsumura et al. 2017). Additionally, Wulff et al. (2017) successfully visualised the interaction between female and male genitalia during copulation in the bushcricket *Metrioptera roeselii* (Hagenbach, 1822) using a radiography technique. These traditional and innovative technologies have provided new insights into genital coupling. However, no single method can supply complete information on genital interactions. Instant fixation unveils the detailed morphology of coupled genitalia in 3D but only as a snapshot, while the radiography technique captures movement but lacks sufficient spatial resolution for smaller insects. Therefore, accumulating data from diverse approaches is essential to achieve a holistic understanding of genital interactions.

**Figure 1.**
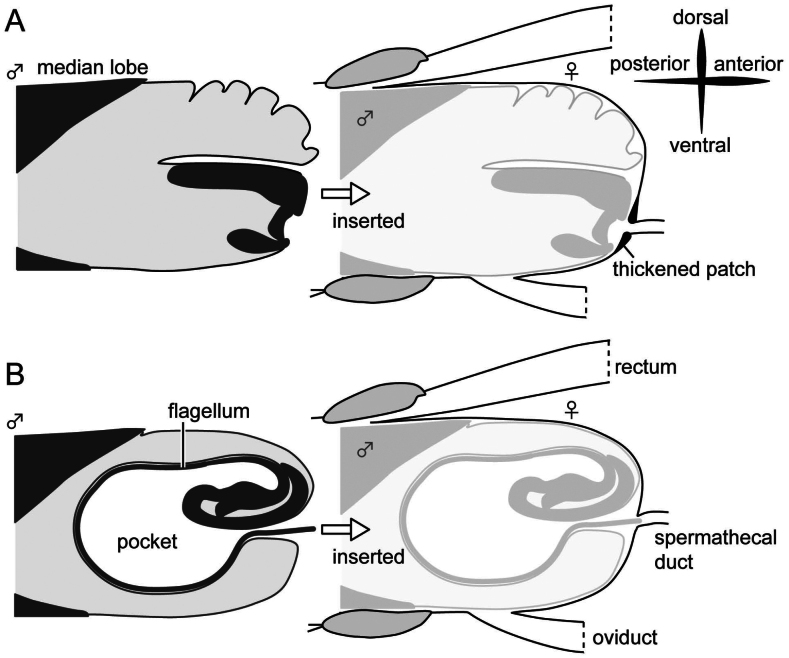
Schemas of female and male genitalia in the subfamily Criocerinae**A** plesiomorphic state of the endophallus and its genital coupling drawn based on Düngelhoef and Schmitt (2006) and Schmitt and Uhl (2015)**B** derived state of the endophallus observed in the subgenus Lema and its genital coupling drawn based on Matsumura and Akimoto (2009) and Matsumura and Yoshizawa (2010); the pocket is formed by an invagination of the endophallus membrane including sclerites (Matsumura et al. 2013). Light grey in the schemas of the male genitalia indicates the body cavity.

The present study focuses on shining leaf beetles (Criocerinae), one of four subfamilies, Criocerinae, Donaciinae, Sagrinae and Bruchinae, that possibly form a monophyletic group (Schmitt 1988; Gomez-Zurita et al. 2008; Nie et al. 2020). The male genital morphology, with a particular focus on the structure involved in genital interaction namely endophallus, and genital coupling have been relatively well-studied in this group (Hayashi 2005; Düngelhoef and Schmitt 2006; Matsumura and Suzuki 2008; Matsumura and Akimoto 2009; Düngelhoef and Schmitt 2010; Matsumura and Yoshizawa 2010, 2012; Schmitt and Uhl 2015; Hayashi 2020; Schmitt et al. 2023). Cricerinae exhibits two types of the endophallus morphology (Fig. 1, left side) referred to as types A and B, with type A considered the plesiomorphic state (Matsumura and Yoshizawa 2012). Type B is characterised by an invagination of the endophallus membrane including sclerites, forming a pocket to accommodate the elongated sperm-transmitting organ, flagellum (Matsumura and Yoshizawa 2012; Matsumura et al. 2013). Parallel evolution of the pocket and flagellum, consisting of various combinations of endophallus sclerites observed in the plesiomorphic state, has been documented in the subfamily (Düngelhoef and Schmitt 2006; Matsumura and Yoshizawa 2012; Matsumura et al. 2014).

Endophallus surfaces have often been examined in taxonomic and morphological studies on this possibly monophyletic group and are frequently covered with comb-like projections and denticles (Düngelhoef and Schmitt 2006, 2010; Matsumura and Yoshizawa 2010; Hayashi 2020; Schmitt et al. 2023). In contrast, the surface of the bursa copulatrix, which comes into physical contact primarily with the endophallus during copulation in the group (Schmitt and Uhl 2015), has largely been neglected. This pilot study aims to document the bursa copulatrix and endophallus of criocerine beetles, with a particular focus on microstructures of female and male membranous elements, using stereomicroscopy and scanning electron microscopy (SEM).

## ﻿Material and methods

### ﻿Study species

The following five species were studied: Lema (Lema) coronata Baly, 1873; Lema (Lema) delicatula Baly, 1873; Lema (Lema) scutellaris (Kraatz, 1879); *Oulema atrosuturalis* (Pic, 1923); and *Crioceris orientalis* Jacoby, 1885. The metadata of samples used in this study are listed in Table 1. The phylogenetic relationships among the study species, based on Matsumura et al. (2014), are presented in Fig. 12.

**Table 1.** Metadata of female and male samples used in the study.

	Female		Male	
	*N*	collection site	*N*	collection site
Lema (Lema) coronata	5	Japan, Shiga pref.	6	Japan, Shiga pref.
Lema (Lema) delicatula	1	Japan, Shikoku	1	Japan, Shikoku
Lema (Lema) scuteralis	4	Japan, Shiga pref. and their offspring (F1)	3	Japan, Shiga pref. and their offspring (F1)
*Oulema atrosuturalis*	1	Japan, Shiga pref.	2	Japan, Shiga pref.
*Crioceris orientalis*	1	Japan, Hokkaido pref.	1	Japan, Hokkaido pref.

### ﻿Morphological investigation

Samples preserved in 70 or 99.5% ethanol were macerated with a 5–10% KOH solution. Genitalia were dissected in distilled water under an Olympus SZ60 stereomicroscope (Olympus Corporation, Tokyo, Japan). Female and male membranous genital elements were spread out on flat adhesive tapes and air-dried to expose the entire surface and clarify the observed locations. SEM images were taken using a JSM-6510 SEM (JEOL Ltd., Tokyo, Japan) in low vacuum mode. All samples were examined at an accelerating voltage of 20 kV.

### ﻿Terminology for genital orientation

The orientation of male genitalia varies depending on the context in which they are observed. Therefore, I used the terminology based on their orientation when the male genitalia are inserted into the female genitalia (Fig. 1).

## ﻿Results

### ﻿Lemini: *Lema*

Figs 2–7

**Figure 2.**
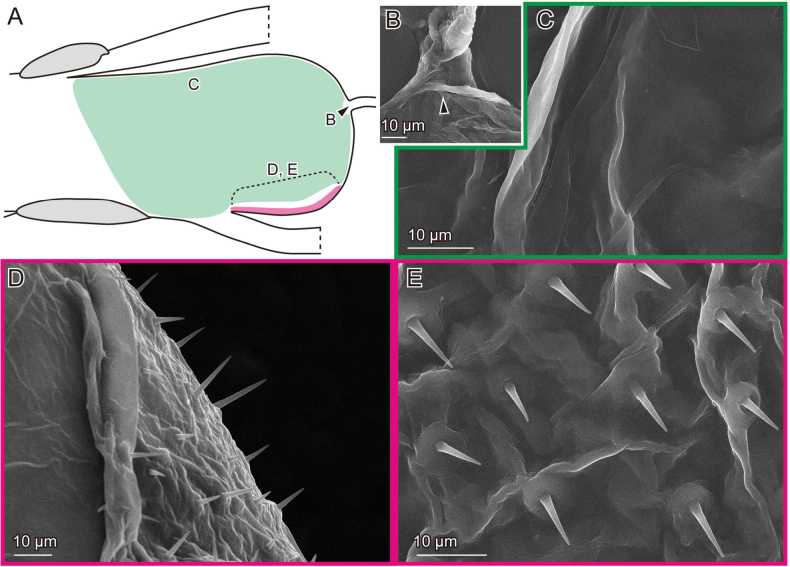
Female genital surfaces of Lema (Lema) coronata**A** schematic drawing of the female genitalia in lateral view; colours and alphabets correspond to those of **B–E**, showing surface microstructures in the inner surface of the corresponding regions. Arrowheads denote the spermathecal duct opening.

The female exhibits similar features across three study species. The spermathecal duct opens directly onto the membrane of the bursa copulatrix (Figs 2B, 4B). The surface of the bursa copulatrix is predominantly smooth (Figs 2A, C, 4A, C), with a sensilla-bearing patch located ventrally to the spermathecal duct opening (Figs 2A, D, E, 4A, C, D, 6). In *L. delicatula*, the only female sample observed was covered with debris, obscuring surface visibility in Fig. 6. The patches do not appear completely membranous, as they exhibit fewer wrinkles compared to other observed regions. In *L. scutellaris*, less prominent microprotrusions are present dorsally (Fig. 4E).

**Figure 3.**
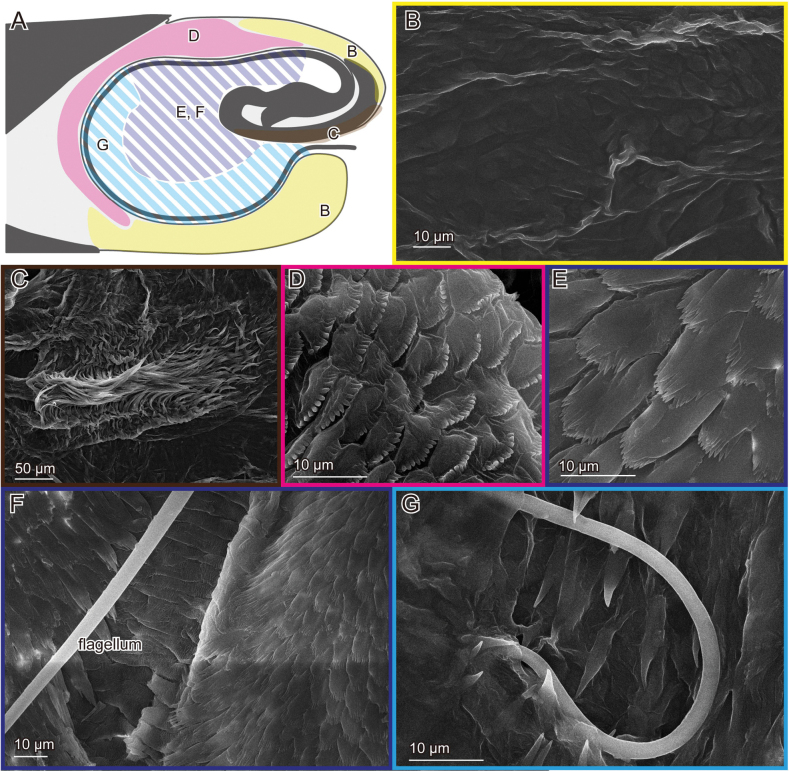
Male genital surfaces of Lema (Lema) coronata**A** schematic drawing of the male genitalia in lateral view; colours and alphabets correspond to those of **B–G**, showing surface microstructures in the corresponding regions; regions with oblique lines show the pocket membrane, and the others show outer surfaces of the endophallus.

In contrast to the female, the surface of the male genitalia is covered with microprotrusions, particularly on the pocket membrane (Figs 3, 5, 7). These microprotrusions are scale-like, with numerous finger-like protrusions pointing toward the pocket fold where the flagellum is housed. The region, where the flagellum lies, bears no microprotrusions (Fig. 3F). The ratio between the scale body and finger-like parts varies across locations: the scale body is larger near the basal part of the flagellum and its exit (Figs 3A, E, F, 5A, G, 7A, B, C), while the finger-like protrusions are prominent at the edge closer to the pocket fold (Figs 3A, G, 5A, H, I, 7A, D). The outer surface is also covered with similar microprotrusions, predominantly in *L. scutellaris* (Fig. 5A–F) and only partially in *L. coronata* and *L. delicatula* (Figs 3A, C, D, 7A, D). In *L. coronata* and *L. delicatula*, possible campaniform sensilla are present. In the former species, those are situated on the postero-dorsal surface and are situated on the postero-ventral surface in the latter species (Figs 5A, D, 7A, E).

**Figure 4.**
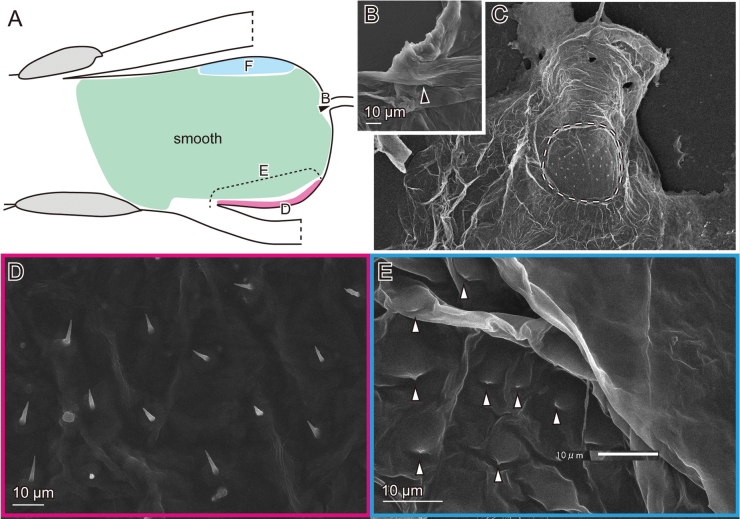
Female genital surfaces of Lema (Lema) scutellaris**A** schematic drawing of the female genitalia in lateral view; colours and alphabets correspond to those of **B–E**, showing surface microstructures in the inner surface of the corresponding regions, except for green one, which is smooth. Black arrowheads denote the spermathecal duct opening, and white ones denote less prominent microprotusion.

**Figure 5.**
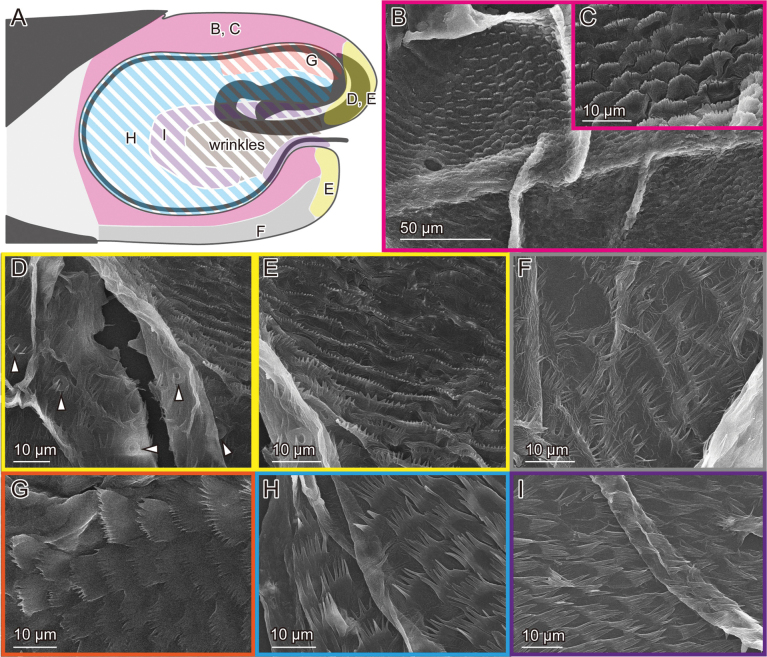
Male genital surfaces of Lema (Lema) scutellaris**A** schematic drawing of the male genitalia in lateral view; colours and alphabets correspond to those of **B–I**, showing surface microstructures in the outer surfaces of the corresponding regions, except for one covered with wrinkles; regions with oblique lines represent the pocket membrane, and the others show the outer surfaces of the endophallus.

**Figure 6.**
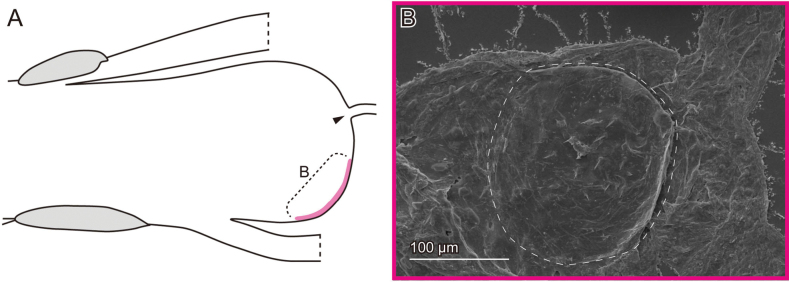
Female genital surfaces of Lema (Lema) delicatula**A** schematic drawing of the female genitalia in lateral view; colour and alphabet correspond to those of **B**, showing surface microstructures in the inner surface of the corresponding region. Arrowhead denotes the spermathecal duct opening. Dashed lines depict the thickened patch.

**Figure 7.**
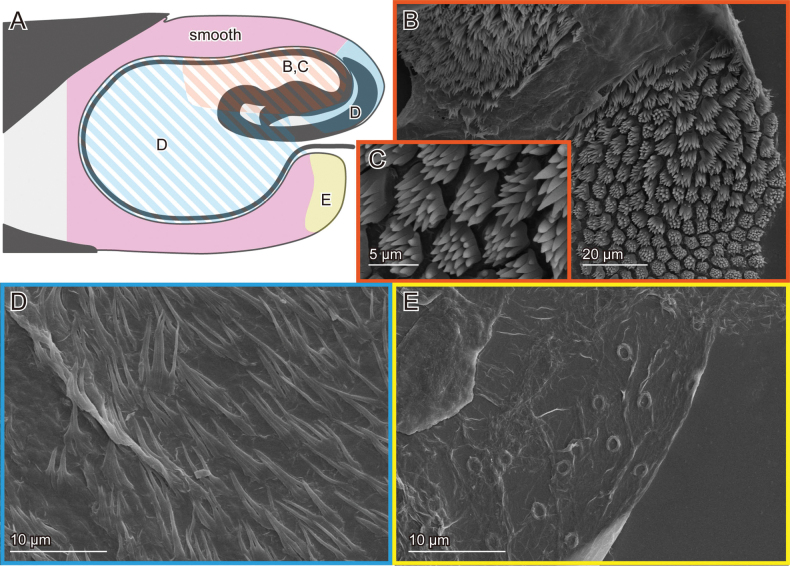
Male genital surfaces of Lema (Lema) delicatula**A** schematic drawing of the male genitalia in lateral view; colours and alphabets correspond to those of **B–E**, except for the red one which is smooth; regions with oblique lines represent the pocket membrane, and the others show the outer surfaces of the endophallus.

### ﻿Lemini: *Oulema*

Figs 8, 9

**Figure 8.**
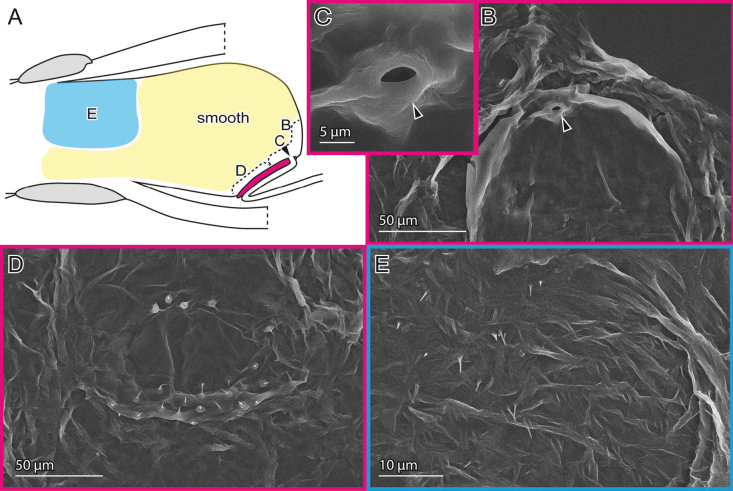
Female genital surfaces of *Oulema atrosuturalis***A** schematic drawing of the female genitalia in lateral view; colours and alphabets correspond to those of **B–E**, showing surface microstructures in the inner surface of the corresponding regions, except for the yellow one which is smooth. Arrowheads denote the spermathecal duct opening.

The female possesses a thickened patch that includes the spermathecal duct opening, which is approximately 5 µm in diameter (Fig. 8B, C). Two clusters of sensilla are situated below the opening (Fig. 8A, D). The spermathecal duct is folded and embedded underneath this patch (Fig. 8A). The dorso-lateral surface is sparsely covered with microprotrusions (Fig. 8A, E).

**Figure 9.**
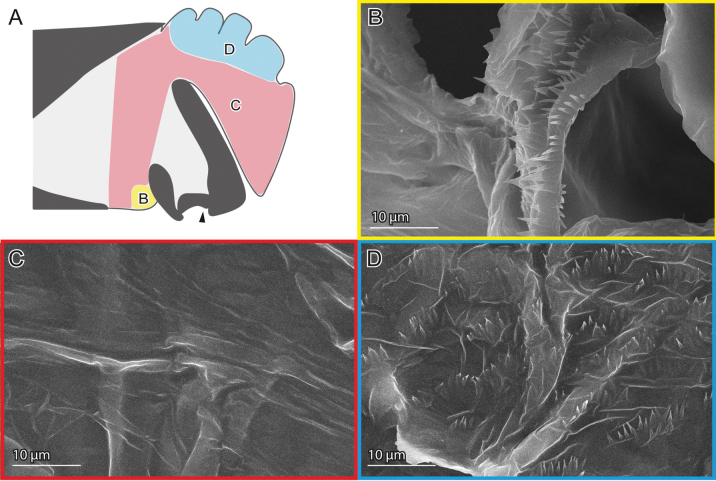
Male genital surfaces of *Oulema atrosuturalis***A** schematic drawing of the male genitalia in lateral view; colours and alphabets correspond to those of **B–D**, showing surface microstructures in the outer surface of the corresponding regions. Arrowhead denotes the gonopore.

The male exhibits the gonopore on the median sclerite (Fig. 9A, arrowhead). Adjacent to the sclerites and on the dorsal side, the endophallus membrane is covered with finger-like microprotrusions (Fig. 9A, B, D), while the remaining surface is smooth (Fig. 9A, C).

### ﻿Criocerini: *Crioceris*

Figs 10, 11

**Figure 10.**
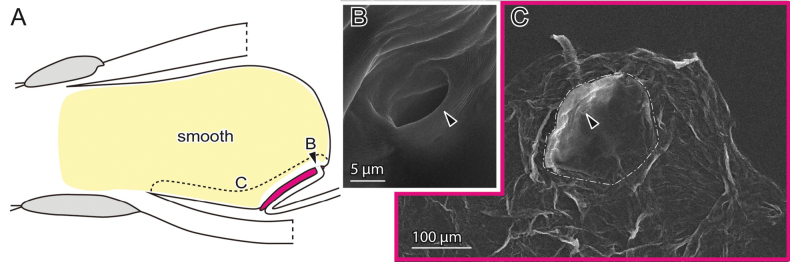
Female genital surfaces of *Crioceris orientalis***A** schematic drawing of the female genitalia in lateral view; colours and alphabets correspond to those of **B, C**, showing surface microstructures in the inner surface of the corresponding regions, except for the yellow one, which is smooth. Arrowheads denote the spermathecal duct opening. Dashed lines depict the thickened patch.

**Figure 11.**
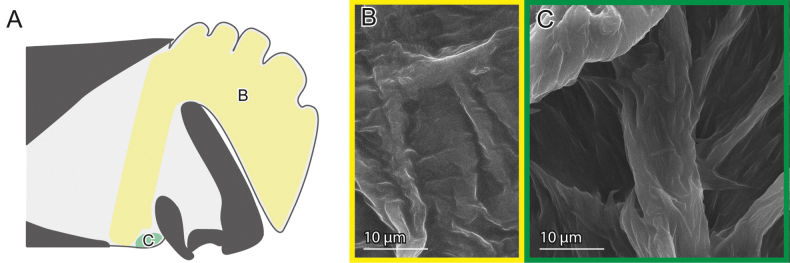
Male genital surfaces of *Crioceris orientalis***A** schematic drawing of the male genitalia in lateral view; colours and alphabets correspond to those of **B** and **C**, showing surface microstructures in the outer surface of the corresponding regions.

The female possesses a slightly thickened patch but lacks sensilla, unlike the other study species (Fig. 10A, C). The spermathecal duct is folded beneath the patch, with its opening at the anterior edge (Fig. 10). Microprotrusions on the genital surface are absent (Fig. 10A). Similarly, the male genital surfaces lack microprotrusions (Fig. 11A, B), while fine wrinkles are present on the ventral surface (Fig. 11A, C).

## ﻿Discussion

**Figure 12.**
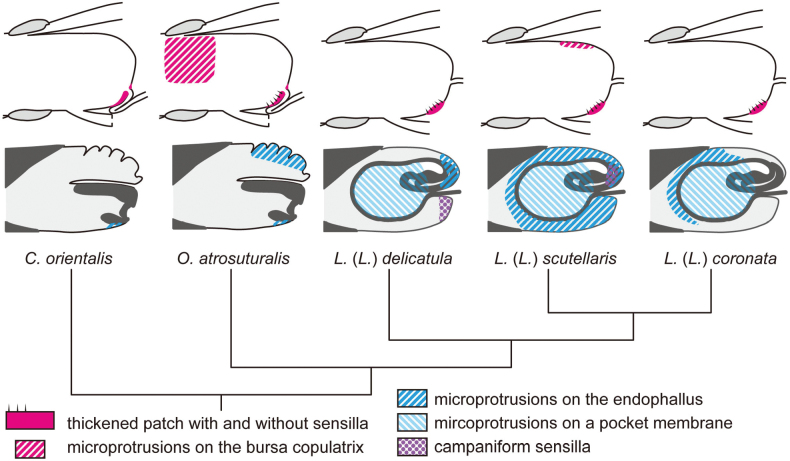
Schematic summary of the obtained results plotted on the phylogenetic tree of the study species based on Matsumura et al. (2014). Upper schemas depict the female genitalia, lower ones show the male genitalia of the study species. The distribution of observed microstructures is mapped on these schemas.

The present study shows that microprotrusions on the bursa copulatrix are less prominent than those on the endophallus across the study species, while in all species except for *C. orientalis*, sensilla were observed either on or dorsal to the thickened patch (Fig. 12). Since those samples were dehydrated under ambient conditions and the thickened patch was covered with no wrinkles, the patch and sensilla are likely more sclerotised than the other membranous regions, even though they are not melanised like typical sclerites. A pointed sclerotised structure is known on the bursa copulatrix in the galerucine species *Metrioidea elongatus* (Jacoby) and is considered a spermatophore opener (Flowers and Eberhard 2006). However, spermatophore formation has not been documented in Criocerinae, and despite thousands of dissections, I have never observed spermatophores in this group. This combination of sensilla and a thickened patch resembles paired sclerotised patches equipped with mechanoreceptive sensilla on the dorsal surface of the vagina in Odonata (Córdoba-Aguilar 2003). In Odonata, these sensilla are campaniform sensilla that detect the presence of an egg in the vagina and trigger sperm release from the spermatheca (Córdoba-Aguilar 2003). This functional possibility may be worth testing in Criocerinae. Another possible function is the stimulation of the endophallus during copulation, as the sensilla touches the endophallus.

Given that the thickened patch itself at the opening of the spermathecal duct was reported also in *Lilioceris* (Berti and Rapilly 1976; Schmitt and Uhl 2015) and in all Japanese donaciine and crioceine species except for the members of the subgenus Lema (Matsumura and Suzuki 2008), the presence of this structure is a plesiomorphic state in Criocerinae. This implies that the separation between the patch and the spermathecal duct opening observed in the subgenus Lema is a derived condition, potentially co-evolved with the acquisition of the flagellum stored within the pocket of the endophallus. If the folded spermathecal duct observed in this study is also a widespread feature within the subfamily, such co-evolution is plausible, as the folded spermathecal duct may impede flagellum penetration. The evolution of the flagellum occurred multiple times in Criocerinae (Düngelhoef and Schmitt 2006; Matsumura and Yoshizawa 2012; Matsumura et al. 2014; Schmitt and Uhl 2015), and this co-evolution hypothesis requires formal testing with broader taxon sampling.

Membranous microprotrusions on the endophallus were observed in all study species except for *C. orientalis*. These microprotrusions are well-known and widespread in Phytophaga (Yokoi 1990; Düngelhoef and Schmitt 2006, 2010; Hubweber and Schmitt 2010; Erbey and Candan 2014, 2015, 2018), although it is often not explicitly mentioned whether they are membranous or sclerotised. Membranous microprotrusions have been reported in other criocerine species, *O. duftschmidi* (Redtenbacher, 1874) and *Lilioceris lilii* (Scopoli, 1763) (Düngelhoef and Schmitt 2006). Düngelhoef and Schmitt (2006) suggested that these microprotrusions serve “to support the mechanical coupling of the mates” and to stimulate females. Sclerotised but similarly shaped microprotrusions covering the endophallus in *Acanthoscelides obtectus* (Say, 1831) (Bruchinae) were hypothesised to cause wounds on the bursa copulatrix (Düngelhoef and Schmitt 2006). However, this hypothesis was rejected for *A. obtectus* (Schmitt et al. 2023), where the structures are considered to increase friction and avoid slipping from the bursal copulatrix (Schmitt et al. 2023).

Given that the surfaces of the bursa copulatrix in Criocerinae are largely smooth, it seems unlikely that microprotrusions increase friction due to the absence of interacting structures. In leaf beetles, pairs remain connected not only through genital coupling but also through male adhesion to the female’s back with their tarsal attachment structures (Voigt et al. 2017; Matsumura et al. 2023). Therefore, I consider the stimulatory function proposed by Düngelhoef and Schmitt (2006) as an alternative function more plausible. Although I observed sensilla on the bursa copulatrix in four out of five study species, the locations of female sensilla and male microprotrusion do not correspond to each other. These microprotrusions could reduce contact surface areas between the bursa copulatrix and endophallus, thereby decreasing adhesion between the two contact surfaces. Therefore, I speculate those microprotrusions may facilitate detachment during escape from predators as another possible function.

Moreover, the observed campaniform sensilla on the endophallus in *L. coronata* and *L. delicatula* were much less prominent than the campaniform sensilla reported for *Lilioceris* (fig. 14 in Düngelhoef and Schmitt 2006). This lower visibility could be attributed to the sample preparation method used in this study, air-drying on a flat tape. Düngelhoef and Schmitt (2006) employed Hexamethyldisilazane (HMDS) drying, which likely retains fine and membranous structures better. Future studies should not only document the morphological features of both female and male genitalia using this appropriate drying method but also investigate the physical properties of the observed microstructures. This can be done relatively easily using a method with confocal laser scanning microscopy, as proposed by Michels and Gorb (2012), for estimating material properties of chitinous structures. For a comprehensive understanding of female and male genital interaction, an extensive survey of the female genital morphology is essential, and the abovementioned functional hypotheses should be rigorously tested.

## ﻿Additional information

### Author ORCID

Yoko Matsumura https://orcid.org/0000-0002-3438-2161

### Data availability

All of the data that support the findings of this study are available in the main text.
